# Impaired Excitatory Drive to Spinal Gabaergic Neurons of Neuropathic Mice

**DOI:** 10.1371/journal.pone.0073370

**Published:** 2013-08-23

**Authors:** Jörg Leitner, Sören Westerholz, Bernhard Heinke, Liesbeth Forsthuber, Gabriele Wunderbaldinger, Tino Jäger, Doris Gruber-Schoffnegger, Katharina Braun, Jürgen Sandkühler

**Affiliations:** 1 Department of Neurophysiology, Center for Brain Research, Medical University of Vienna, Vienna, Austria; 2 Institute of Physiology, Faculty of Medicine, Otto von Guericke University Magdeburg, Magdeburg, Germany; 3 Institute of Biology, Faculty of Natural Sciences, Otto von Guericke University Magdeburg, Magdeburg, Germany; University of Arizona, United States of Ameirca

## Abstract

Adequate pain sensitivity requires a delicate balance between excitation and inhibition in the dorsal horn of the spinal cord. This balance is severely impaired in neuropathy leading to enhanced pain sensations (hyperalgesia). The underlying mechanisms remain elusive. Here we explored the hypothesis that the excitatory drive to spinal GABAergic neurons might be impaired in neuropathic animals. Transgenic adult mice expressing EGFP under the promoter for GAD67 underwent either chronic constriction injury of the sciatic nerve or sham surgery. In transverse slices from lumbar spinal cord we performed whole-cell patch-clamp recordings from identified GABAergic neurons in lamina II. In neuropathic animals rates of mEPSC were reduced indicating diminished global excitatory input. This downregulation of excitatory drive required a rise in postsynaptic Ca^2+^. Neither the density and morphology of dendritic spines on GABAergic neurons nor the number of excitatory synapses contacting GABAergic neurons were affected by neuropathy. In contrast, paired-pulse ratio of Aδ- or C-fiber-evoked monosynaptic EPSCs following dorsal root stimulation was increased in neuropathic animals suggesting reduced neurotransmitter release from primary afferents. Our data indicate that peripheral neuropathy triggers Ca^2+^-dependent signaling pathways in spinal GABAergic neurons. This leads to a global downregulation of the excitatory drive to GABAergic neurons. The downregulation involves a presynaptic mechanism and also applies to the excitation of GABAergic neurons by presumably nociceptive Aδ- and C-fibers. This then leads to an inadequately low recruitment of inhibitory interneurons during nociception. We suggest that this previously unrecognized mechanism of impaired spinal inhibition contributes to hyperalgesia in neuropathy.

## Introduction

**Figure 1:**
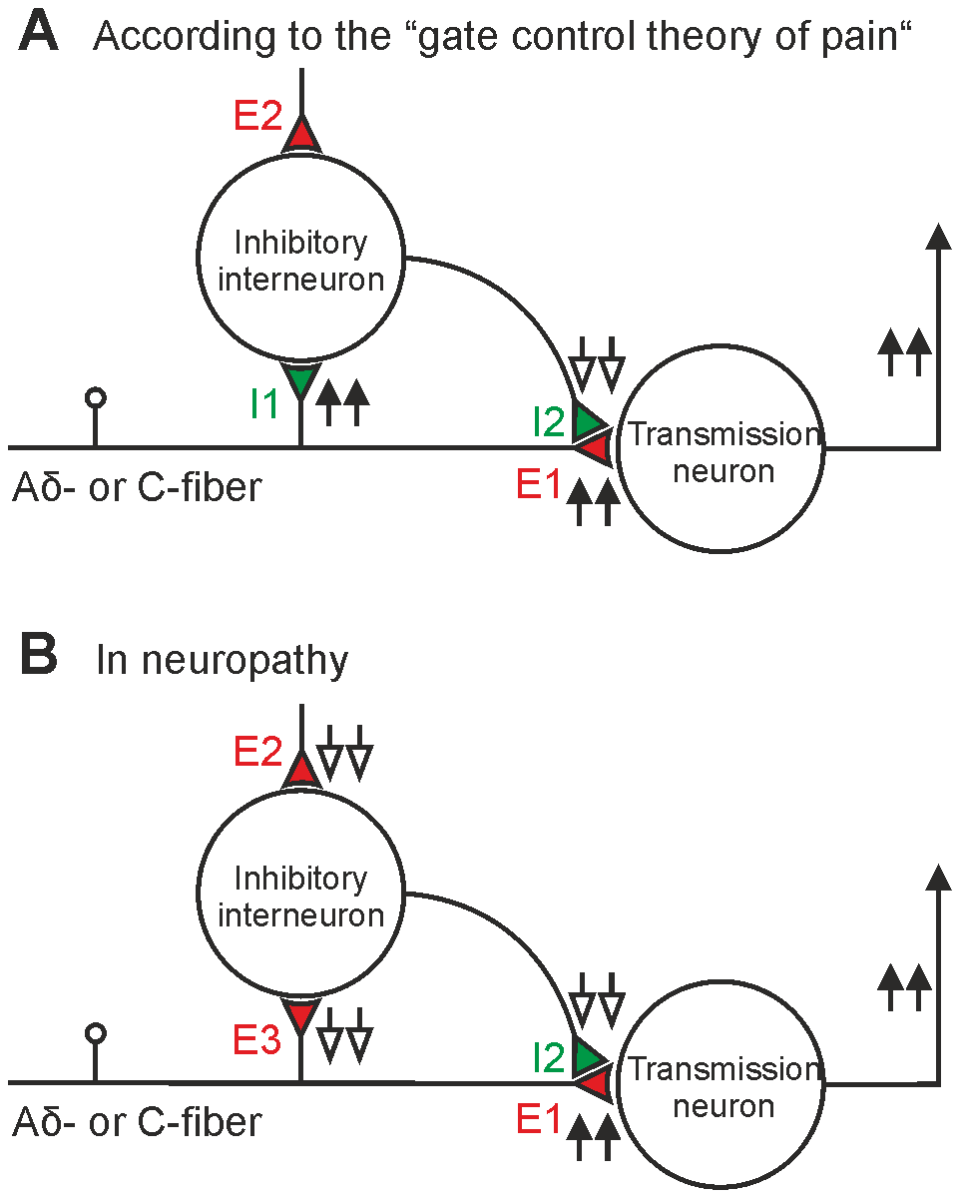
Opening a spinal gate for nociception. (**A**) The classical gate control theory: Afferent nociceptive fibers (Aδ- and C-fibers) directly excite transmission neurons via excitatory synapse E1. Collaterals of nociceptive afferents inhibit inhibitory neurons through a proposed inhibitory synapse I1. Activity in Aδ- and C-fibers during nociception would thus depress activation of the inhibitory interneurons. This would lead to a reduced (↓↓) pre- (I2) or postsynaptic inhibition (not shown) of nociceptive transmission neurons in the spinal dorsal horn. The existence of the proposed inhibitory synapse (I1) has, however, not been shown experimentally. (**B**) The present and previous studies [10], [16] have demonstrated monosynaptic excitatory input (E3) to GABAergic neurons from Aδ- and C-fiber afferents. We tested the hypothesis, that in neuropathy global excitatory drive (E2 and E3) including direct excitation from presumably nociceptive Aδ- and C-fibers to GABAergic neurons is impaired. This would lead to a reduced inhibition at I2 (↓↓) and thus open the spinal gate for nociception (↑↑ E1). E2: other excitatory input from local circuits or descending pathways. (color code: green: inhibitory synapse; red: excitatory synapse)

Impaired GABAergic inhibition in the spinal cord may contribute to neuropathic pain which is characterized by increased sensitivity to thermal and mechanical stimuli (thermal and mechanical hyperalgesia) [1], [2]. Blockade of GABAergic inhibition in the spinal cord mimics some symptoms of neuropathic pain [3], and activation of spinal GABA receptors can alleviate neuropathic pain in animals [4] and in humans [5]. The mechanisms underlying impaired GABAergic inhibition in the spinal cord in neuropathy are not fully understood but a number of different models have been proposed [6]–[8]. In their classical “gate control theory of pain” Melzack and Wall [9] suggested that inhibitory interneurons in the superficial spinal dorsal horn “function as a gate control system”. Nociceptive afferent fibers would directly depress these inhibitory neurons which “holds the gate in a relatively open position”. Thus, the “output of the T(ransmission) cell rises” [9] (see Fig. 1A). Experimental evidence for a direct depression of inhibitory interneurons by nociceptive nerve fibers is, however, lacking as nociceptive afferent fibers excite, rather than depress spinal GABAergic neurons [8], [10]. Here we tested a different, but closely related hypothesis. We speculated that under conditions of a neuropathy the excitatory drive to inhibitory GABAergic neurons might be reduced. A reduced excitation would functionally be equivalent to an increase in depression of inhibitory neurons and could thus equally well open a gate for nociceptive messages to the brain (Fig. 1B) and thus lead to mechanical and thermal hyperalgesia.

## Methods

### Animals

Mice were treated in strict accordance with directive 2010/63/EU of the European Parliament and of the council of the EU. The protocol was approved by the Austrian Federal Ministry of Science and Research (Permit Number: 66.009/097-C/GT/2007). All surgery was performed under isoflurane anesthesia, and all efforts were made to minimize suffering.

Male, 8 – 10 weeks old, homozygous transgenic mice, expressing EGFP under the control of the promoter for GAD67 (GIN mice, [11]) (Jackson Laboratories, USA; strain name: FVB-TgN (GadGFP)45704Swn) were used in this study. Animals were kept and interbred in local facilities with food and water supply *ad libitum*.

### Nerve ligation

Animals underwent either a sham surgery or a modified version [8] of chronic constriction injury (CCI) surgery, a model for neuropathic pain [12]. Mice were deeply anesthetized with isoflurane and the left sciatic nerve was exposed at the mid-thigh level. Proximal to the trifurcation the nerve was freed of adhering tissue and three ligatures (7–0 prolene) were tied around it with about 1 mm spacing in between. Ligatures were tied until they elicited a twitch in the hind paw. Muscle and skin incision were then closed. Sham-treated mice, that underwent the same procedure except ligation, were used as control animals.

### Behavioral tests

Mechanical thresholds were assessed with calibrated von Frey monofilaments with incremental stiffness (Stoelting, USA) according to the up-and-down method [13]. The 50% threshold was calculated, which indicates the force of von Frey hair at which an animal reacts in 50% of the presentations [14].

Thermal nociceptive thresholds were determined by responses to a radiant heat source focused on the plantar surface of the rats hindpaw with a Plantar Test Instrument (Ugo Basile, Italy) [15]. The paw withdrawal latency was recorded by a digital timer with a cut-off of 30 s.

### Spinal cord slice preparation

Ten to 13 days after surgery mice were anesthetized with isoflurane and the lumbar spinal cord was removed. The spinal cord was transferred to cold (∼4°C), oxygenated incubation solution composed of (mM): NaCl 95, MgSO_4_ 7, CaCl_2_ 0.5, KH_2_PO_4_ 1.2, KCl 1.8, NaHCO_3_ 26, glucose 15, sucrose 50, oxygenated with 95% O_2_, 5% CO_2_; pH 7.4, measured osmolarity 305 – 320 mosmol·l^−1^. After removal of all roots except dorsal roots L4 – L6 (ipsilateral to the surgery), the spinal cord was cut on a DSK microslicer (DTK-1000, Dosaka, Japan) into ∼500 µm thick transverse spinal cord slices with a dorsal root attached. For two-photon laser-scanning microscopy parasagittal lumbar spinal cord slices were prepared on the microslicer at ∼300 µm thickness. After preparation spinal cord slices were transferred to 30°C incubation solution and kept there for at least 1 h.

### Electrophysiology

Single transverse slices were transferred to a recording chamber and superfused with oxygenated recording solution at 3 ml·min^−1^ at 33°C. The recording solution was identical to the incubation solution except for (in mM): NaCl 127; CaCl_2_ 2.4; MgSO_4_ 1.3 and sucrose 0. Lamina II dorsal horn neurons were visualized with infrared light using a cooled CCD camera (PCO, Germany). EGFP expressing neurons were identified by epifluorescence microscopy. Neurons located within a distance of 20 to 100 µm from the dorsal white matter were regarded as lamina II neurons [16].

Whole-cell voltage-clamp experiments were performed with glass pipettes (2.5 – 4.5 MΩ) containing an internal solution consisting of (mM): potassium gluconate 120, KCl 20, MgCl_2_ 2, Hepes 20, Na_4_EGTA 0.5, Na_2_ATP 2, NaGTP 0.5, pH 7.28 adjusted with KOH, measured osmolarity 300 mosmol·l^−1^. For blockade of intracellular Ca^2+^ ions the internal solution was modified for (mM): BAPTA-K_4_ 20 and potassium gluconate 80. Recordings were realized with a multiclamp amplifier (Axopatch 700 B) and the pCLAMP 9 or 10 acquisition software (Molecular Devices, USA). Recordings were low-pass filtered at 2–10 kHz and sampled at 5–100 kHz.

### mEPSC recordings

Miniature excitatory postsynaptic currents (mEPSCs) were measured in the presence of tetrodotoxin (TTX, 1 µM), D-2-amino-5-phosphonovaleric acid (D-AP5, 50 µM; both Ascent Scientific), strychnine (4 µM) and bicuculline (10 µM; both Sigma-Aldrich) from minute 6 – 10 after establishing the whole-cell configuration. At this time interval following whole-cell conformation establishment mEPSC frequency had stabilized. Clamp potential was set at –75 mV. Series resistance was controlled before and after mEPSC-recordings. Only cells with stable series resistances ≤ 30 MΩ were used for analysis.

When the action of agonists and antagonists on mEPSCs was investigated, mEPSC recordings from minute 6 – 10 after establishing the whole-cell configuration were used as a baseline. Drugs were added at minute 10 and mEPSC recording was performed at least until minute 20 after establishing the whole-cell configuration. Bath applied drugs were the CB_1_ antagonist 1-(2,4-Dichlorophenyl)-5-(4-iodophenyl)-4-methyl-N-(piperidin-1-yl)-1H-pyrazole-3-carboxamide (AM 251, 5 µM), the CB_1_ agonist (5Z,8Z,11Z,14Z)-N-(2-Chloroethyl)icosa-5,8,11,14-tetraenamide (ACEA, 1 µM), the NO-donor S-Nitroso-N-acetyl-DL-penicillamin (SNAP, 200 µM, all from Ascent Scientific), and γ-Aminobutyric acid (GABA, 10 µM, Sigma-Aldrich). Control mEPSC recordings were performed without drug application.

Data analysis was done offline by an investigator blinded to the treatment groups using MiniAnalysis Software (Synaptosoft, USA). Miniature postsynaptic currents were first detected automatically by the software using an amplitude threshold of 10 pA and an area threshold of 15 fC. All events were then visually reexamined. Any noise that spuriously met the trigger specifications was rejected (see also: [17]).

### Paired-pulse recordings following dorsal root stimulation

Dorsal roots were stimulated via a suction electrode connected to a constant current stimulus isolator (A 360R, WPI, USA) at 0.1 ms pulse width. Excitatory postsynaptic currents (EPSCs) were recorded in the presence of strychnine (4 µM) and bicuculline (10 µM) and classified to be either Aδ- or C-fiber-evoked according to their latency and threshold. Constant latencies and absence of failures during 10 Hz stimulation (for Aδ-fibers) or 1 Hz stimulation (for C-fibers) were used as criteria for monosynaptic transmission [18]. Thresholds of stimulation intensity of Aδ- and C-fibers did not differ significantly between sham- and CCI-treated mice: Aδ-fibers (sham: 203±32 µA, CCI: 258±44 µA), C-fibers (sham: 1.7±0.2 mA, CCI: 2.1±0.2 mA). The stimulation intensity was set to 200% of threshold values. Paired stimuli were applied at an interstimulus interval of 50 ms, 300 ms and 500 ms, respectively. Pairs of pulses were delivered at an interval of 15 s. For analysis at least 10 traces were averaged, the amplitudes of the first (P1) and the second (P2) EPSC were measured and the paired pulse ratio (PPR) was calculated as PPR  =  P2•P1^−1^.

### Immunohistochemical detection of c-Fos

Tissue was prepared 11 days after sham- or CCI treatment. Mice were briefly anesthetized with isoflurane and the hindpaw of the operated side was immersed into 52°C hot water for 20 seconds to induce c-Fos expression in spinal cord dorsal horn. Two hours later mice were deeply anesthetized with isoflurane and perfused with 0.9% saline followed by 4% paraformaldehyde (PFA) in 0.1 M phosphate buffer (PB) (pH 7.4). Spinal cords were removed, postfixed in the same fixative overnight, cryoprotected by immersion in 20% sucrose in 0.1 M PB for 48h at 4°C and frozen in isopentane at –80°C.

Beginning from L6, the whole lumbar spinal cord was cut in transverse sections on a freezing microtome (CM 3050; Leica, Germany) at a thickness of 7 µm. Sections were serially mounted onto SuperFrost Plus slides (Menzel, Germany) and air dried.

Sections were incubated overnight with the primary antibodies mouse anti-NeuN (Chemicon, 1∶1500) and rabbit anti-c-Fos (Santa Cruz Biotechnology, 1∶1000). The antibodies were diluted in 5% normal donkey serum (NDS) in 0.1 M PBS containing 0.15% TritonX-100 (PBST).

Secondary antibodies used for immunofluorescence were all raised in donkey. Rinsed sections were incubated for 3 h at room temperature with anti-rabbit IgG conjugated to Cy5 and anti-mouse IgG conjugated to Cy3 (Jackson ImmunoResarch, USA; 1:200 in PBS). Each second series of slides was incubated with anti-rabbit IgG conjugated to Cy3 (only c-Fos).

After washing sections were covered with glass slides in a glycerol based medium containing Mowiol 4-88 (Calbiochem) and Propyl gallate (Sigma-Aldrich) to prevent fading.

Sections were viewed and photographed with a fluorescence microscope (Olympus BX51) equipped with a CCD camera (Olympus DP50) and measurements were performed with analySIS-Software (Soft Imaging Systems, Germany).

Digital images of the ipsilateral dorsal horn of the fourth lumbar segments (L4) were acquired with the 20x objective for neurons (NeuN), GABAergic cells (EGFP) and c-Fos. Quantification was accomplished in 10 to 16 randomly chosen sections of L4 at least 40 µm apart from one another.

### Immunohistochemical detection of PSD-95, GAD67 and Synaptophysin

Eleven days after sham- or CCI treatment the mice were transcardially perfused as described above. The spinal cord was dissected out, postfixed for 4 hours, and after cryoprotection overnight snap frozen in isopentane at –80°C. Per animal four (5 µm thick) transverse sections of the L4 segment were cut on the cryomicrotome, mounted onto glass slides and air dried. For the staining of PSD-95 a modified antigen retrieval [19] by incubating the sections for 2 min at 37°C with Pepsin (Dako, Denmark) was necessary. After rinsing in PBS and blocking in 0.1 M PBS containing 0.1% Tween 80 for 30 min, sections were incubated over night at 4°C in the following primary antibodies: goat anti-PSD-95 (Abcam, 1∶100) and mouse anti-GAD67 (Chemicon, 1∶1000), diluted in blocking medium. Rinsed sections were incubated for 1 hour in rabbit anti-goat IgG conjugated to Cy5 (1∶100) and rabbit anti-mouse IgG conjugated to Cy2 (1∶200). Both secondary antibodies were purchased at Jackson Immuno Research. After rinsing, sections were incubated in monoclonal mouse anti-Synaptophysin labeled with Oyster® 550 (1∶1000, Synaptic Systems) for 1 hour. Coverslipping of the sections was performed as mentioned above.

Four sections from each animal were used for image acquisition. In each section two imaging windows (format: 1024×1024 pixel, zoom factor: 5, image size: 46.89 µm×46.89 µm) within lamina II of the spinal cord dorsal horn were scanned ipsi- and contralateral to the surgery side, respectively. Confocal settings (laser power, gain and offset) were identical for all scans. Images were three times line averaged and scanned sequentially (in order to avoid fluorescent bleed-through) with a 63x oil-immersion lens at a z-separation of 0.3 µm on a Leica TCS SP5 confocal microscope. Pinhole was set at 1 Airy unit (100.26 µm), generating a calculated section thickness of 0.772 µm.

Analysis was performed by an experimenter blind to the treatment groups using ImageJ software (National Institutes of Health, USA). Images were processed by the rolling-ball algorithm at a radius of 10 pixels to subtract background. We used two different approaches to quantify colocalization of the three markers GAD67, PSD-95 and synaptophysin in superficial spinal dorsal horn. To perform a pixel intensity spatial correlation analysis we calculated Mander’s colocalization coefficients pairwise for all three channels after automatic thresholding [20] using the JACoP plugin of ImageJ. The second approach was to generate a mask for each channel with automatic thresholds. The three masks were combined pairwise by Boolean operations. In addition the overlay of all three markers was quantified. We measured the overlaying areas in relative numbers as a function of the total signal area.

### Two-photon laser-scanning microscopy

For two-photon laser-scanning microscopy GABAergic neurons were identified by epifluorescence and patch-clamped in the whole-cell configuration as described above. Since the EGFP-labeling did not fully access all dendritic branches and spines, the fluorescent dye calcein (1 mM, Invitrogen, USA) was added to the internal solution. The fluorescent dye was allowed to diffuse 10 minutes into the neurons before imaging. Imaging was performed on a two-photon laser-scanning microscope consisting of a Leica DM LFS A microscope (Leica, Germany) and a femtosecond Ti-sapphire laser (Chameleon-XR, Coherent, Germany). Excitation light (wavelength 800 nm) was focused by a 63x water immersion objective (0.9 NA). Scanning and image acquisition were controlled with Leica Confocal Software (LCS v.2.61). Emitted light was collected by non-descanned detectors. 3D volume samples of selected segments of the neurons were scanned, depending on the neuron, in areas from 1024×256 pixels to 1024×1024 pixels, at 0.3 µm distance in z-direction.

### Three dimensional analysis of dendritic spines

Spine analysis was performed using a 3D reconstruction software (described in detail in [21], [22]) which allows measuring the geometric parameters of dendritic spines from confocal microscopic image stacks. The basic approach is the sequential comparison between the raw microscope image and an estimated image of a geometric model including dendrites and spines. The optimization process is repeated until an optimal match is achieved. Unbranched dendritic segments were selected from confocal image stacks and a 3-D median filter was used to reduce overall image noise. In contrast to the standard step by step approaches, where deconvolution is applied to compensate degradation caused by the point spread function of the microscope prior to reconstructing the spines, our system applied a vector-based parametric geometric model in order to reduce the number of free parameters. The deconvolution was included as a later step during the model fitting process.

First, a raw model, including roughly estimated center axes for both, the dendritic segment and all protruding spines, was initiated. Each center axis was approximated by a set of model points interconnected by linear interpolation. The radius along this estimated axis was approximated by local radii, which were set at the level of each model point and also interconnected by linear interpolation. The tracking of dendrites and spines was based on a growing model approach. Starting with an initial element of at least two model points, which were positioned by the observer for each dendrite and each spine, the axes “grow” by adding new elements. Tracking was performed under visual control and interactive corrections were allowed at all stages of the tracing process.

In the final step the model was readjusted for an optimized match to the original raw microscope image. A convolved image of the raw model was generated by sampling the model with the same resolution as the microscope image and convolving it by the microscope point spread function, which resulted in a simulated microscope image. By iterative comparison of this image with the original microscope image the model parameters were adjusted and the best fitted model was then used for data analysis. The following parameters were calculated from the adapted geometric model to measure the shape of dendritic spines: volume, surface, length and averaged diameter. In addition, the density of spines was calculated for each dendritic segment.

### Statistics

Analysis of the data was performed using SigmaPlot 11 (Systat). All values are means ± s.e.m (if not stated otherwise). Unpaired Student’s t-test (two-tailed), the non-parametric Mann-Whitney rank sum test, the one-way analysis of variance (ANOVA) and the two-way ANOVA, followed by Tukey’s post-hoc test, were used for statistical comparison where appropriate. The significance level was set at 0.05.

## Results

### Behavior

**Figure 2:**
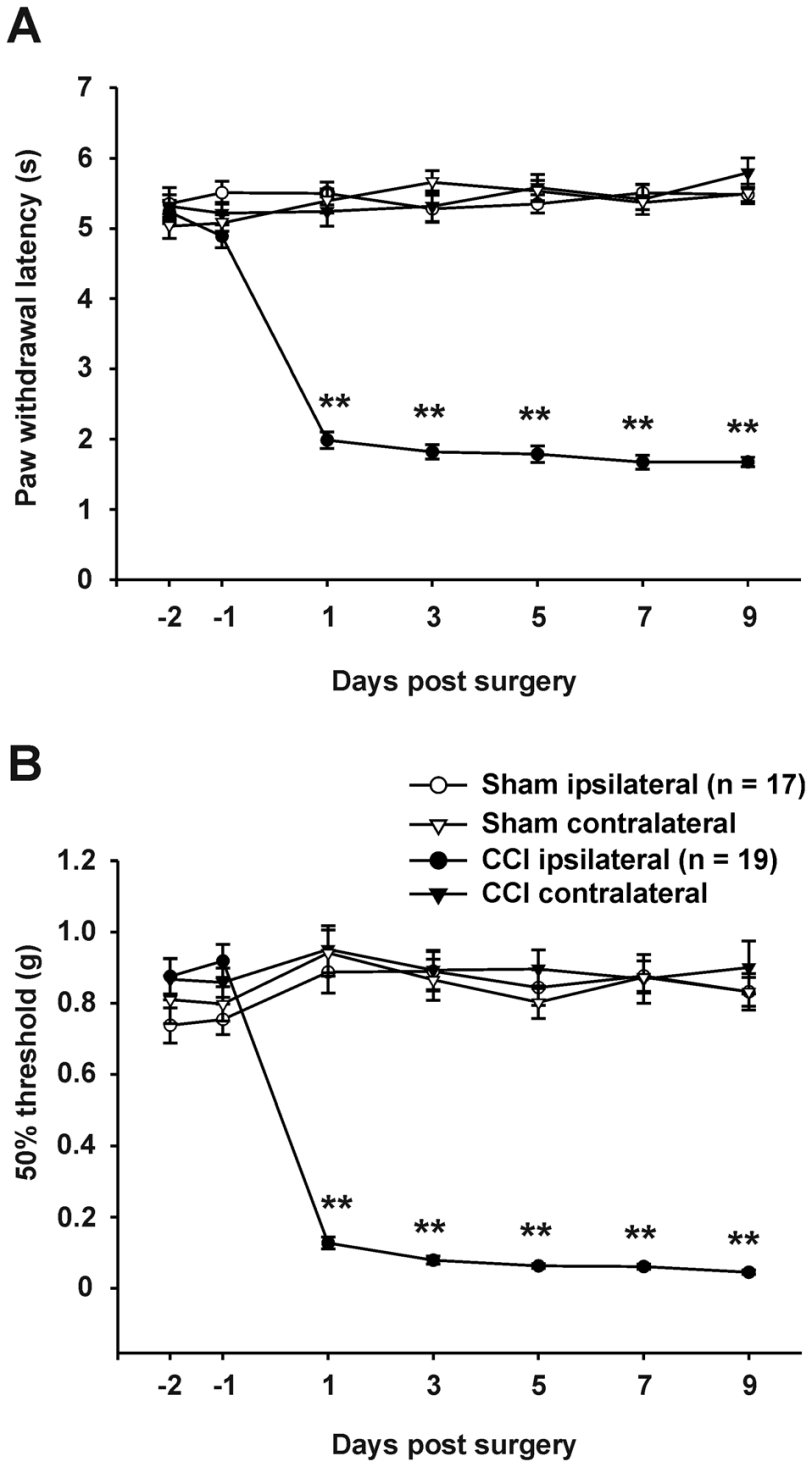
Time course of thermal (A) and mechanical (B) withdrawal thresholds after CCI. Withdrawal thresholds in CCI (n = 19) and sham-operated (n = 17) animals for both ipsi- and contralateral hindpaws. Tests were performed on days 2 and 1 before operation and on days 1, 3, 5, 7 and 9 after surgery. (**A**) Thermal hyperalgesia is indicated by a significant reduction in the paw withdrawal latency. (**B**) Mechanical hyperalgesia is indicated by a significant reduction of the 50% paw withdrawal threshold. Data are expressed as mean ± s.e.m. (* P < 0.05; ** P < 0.01, two-way ANOVA).

All tested CCI-operated but none of the tested sham treated mice expressed thermal (Fig. 2A) and mechanical hyperalgesia (Fig. 2B) throughout the observation period of 9 days.

### mEPSC rate but not amplitude is reduced in CCI-treated animals

**Figure 3:**
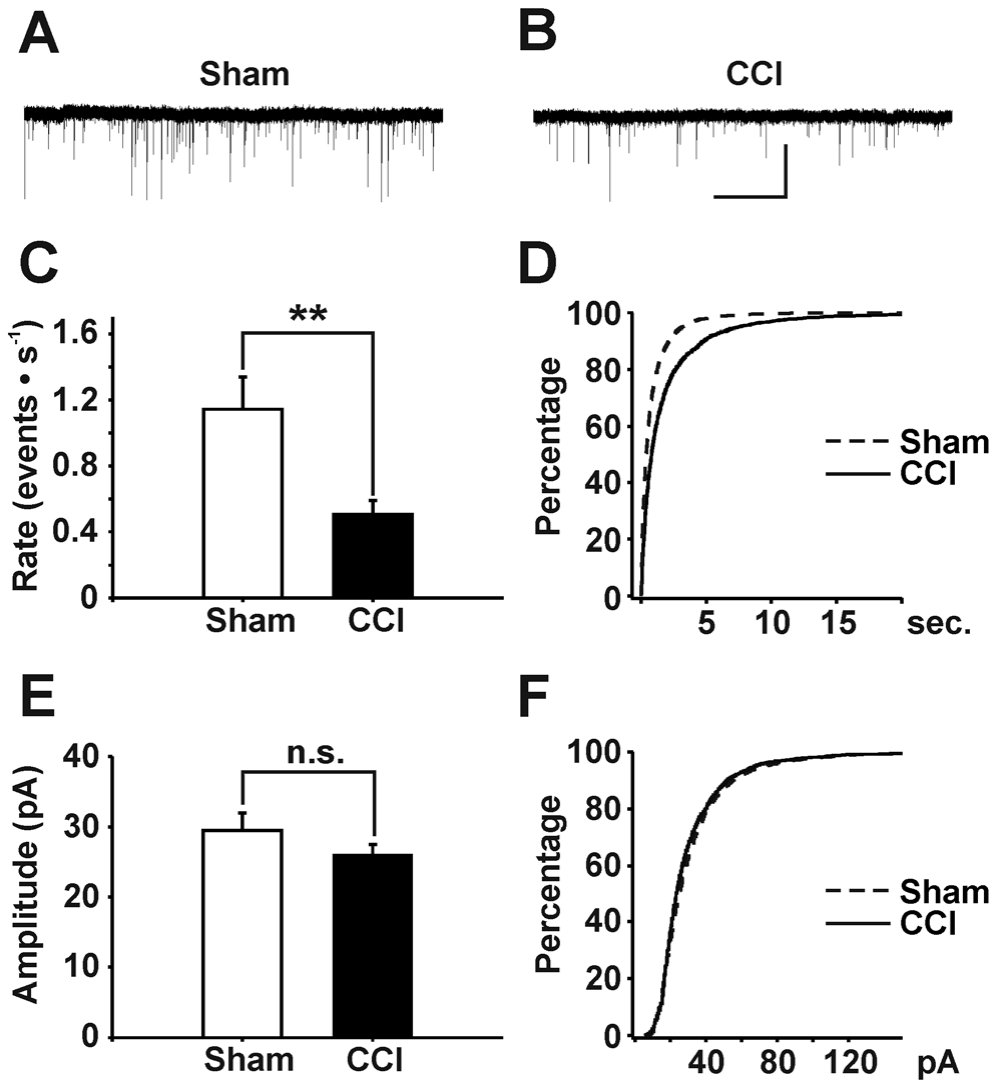
Effects of CCI on mEPSC rates and amplitudes recorded from EGFP-labeled, GABAergic neurons. Representative traces of mEPSCs at a holding potential of -75 mV in sham- (**A**) and CCI-treated (**B**) animals (scale bar: 10 s; 50 pA). (**C**) CCI treated mice exhibited a reduced rate in mEPSCs recorded from GABAergic neurons (n = 34) as compared to sham-operated animals (n = 26 neurons; *** P < 0.001, Mann-Whitney rank sum test). Data: mean ± s.e.m. (**D**) Corresponding cumulative probability plot of inter-event-intervals of mEPSCs. (**E**) Amplitudes of mEPSCs were not significantly changed in CCI treated mice (n = 34 neurons) as compared to sham control (n = 26 neurons, P>0.1, Mann-Whitney rank sum test). Data: mean ± s.e.m. (**F**) Corresponding cumulative probability plot of mEPSC amplitudes.

We assessed the global excitatory input to identified GABAergic neurons in control (Fig. 3A) and in neuropathic animals (Fig. 3B). We recorded mEPSCs from EGFP-labeled GABAergic neurons in spinal cord lamina II. The mean rate (events · s^−1^) of mEPSCs recorded from EGFP-labeled neurons in sham treated mice was 1.14±0.19 (n = 26). In the CCI treated group rate of mEPSCs was significantly lower (0.51±0.08, n = 34, P < 0.001, Fig. 3C and D). In contrast the mean EPSC amplitude was not significantly different in sham treated animals (29.5±2.5 pA) as compared to CCI-operated mice (26.0±1.5 pA, P>0.1, Fig. 3E and F).

Two mechanisms could well explain this decrease in mEPSC rate: a reduced number of synapses or a decrease of neurotransmitter release probability from presynaptic terminals. These two possibilities were explored next.

### CCI-surgery does not change the density or morphology of dendritic spines of GABAergic lamina II interneurons nor the colocalization of GAD67 with markers of excitatory synapses

**Figure 4:**
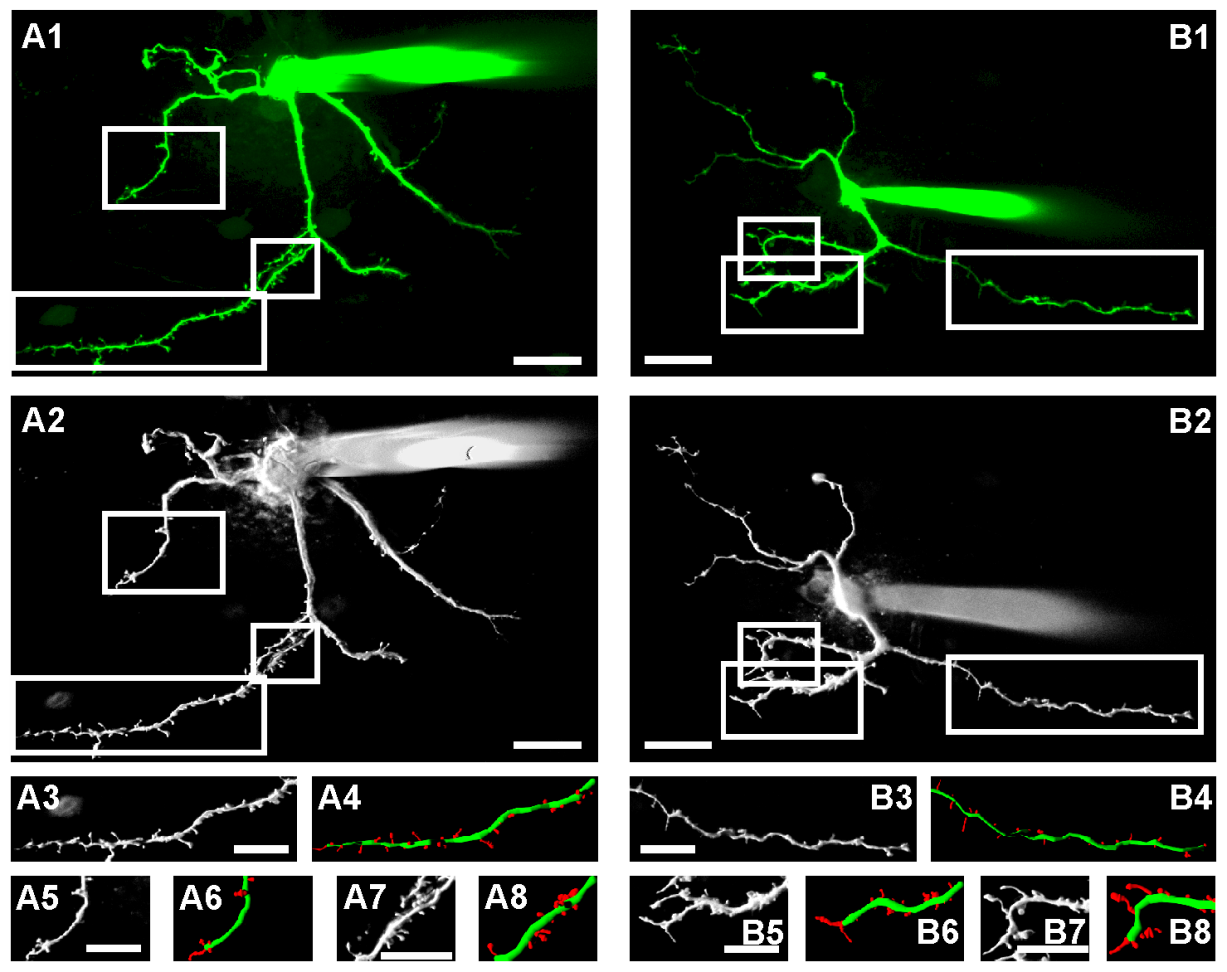
3D reconstruction of dendritic spines of GABAergic neurons. (**A, B**) Representative examples of calcein filled GABAergic neurons from a sham-operated mouse (**A**) and a CCI-operated mouse (**B**). (**A1, B1**) 2D projection of original images of filled neurons (scale bars: 20 µm, left  =  caudal, right  =  rostral). (**A2, B2**) Shadow projection of same neurons. (**A3, A5, A7, B3, B5, B7**) dendritic segments at higher magnification (scale bars: 5 µm). (**A4, A6, A8, B4, B6, B8**) color coded reconstruction of dendritic spines (red) for morphological analysis (scale bars: 5 µm).

We first tested if the number of excitatory synapses converging onto GABAergic neurons might be lower in CCI-animals. Dendritic spines are the predominant locations of excitatory synapses [23], [24]. Their shape correlates with the functional state of a synapse [25]. As an estimate for the number of excitatory synapses of GABAergic neurons we used two-photon laser-scanning microscopy to image and analyze 12 neurons in lamina II from CCI-treated mice and 13 neurons from a sham-treated control group (Fig. 4). Neither the density nor morphological parameters of dendritic spines in GABAergic neurons were significantly different between groups (Table 1).

**Table 1:** Density and morphological parameters of dendritic spines on GABAergic neurons.

	Density (spines per 10 µm)	Length (µm)	Diameter (µm)	Surface (µm^2^)	Volume (µm^3^)
Sham	2.46±0.25	3.10±0.29	0.75±0.06	10.1±1.7	1.93±0.37
CCI	2.58±0.21	2.97±0.21	0.84±0.06	12.1±1.6	2.64±0.44

Neither density nor morphological parameters of dendritic spines on GABAergic neurons were significantly different between sham-treated (n = 12) and CCI (n = 13) animals (Mann-Whitney rank sum test, for all groups: P>0.1). Data: mean ± s.e.m.

**Figure 5:**
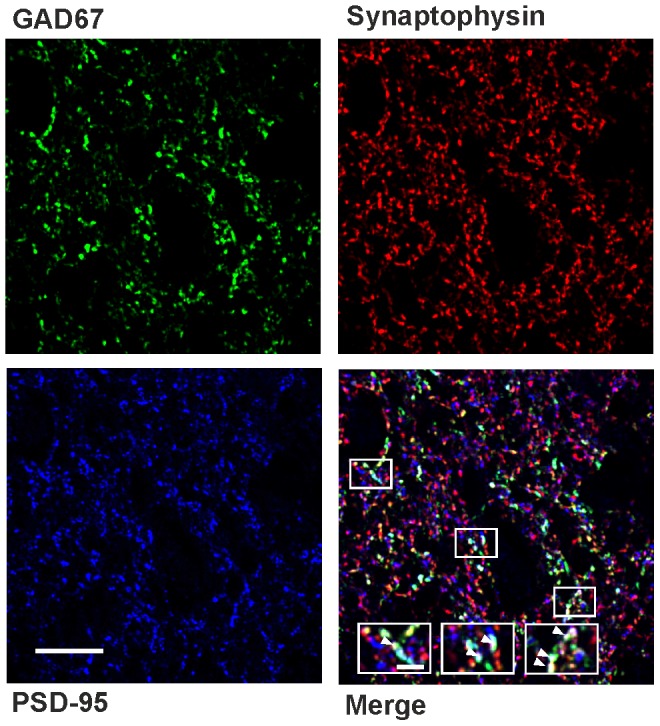
Immunohistochemical triple stainings to quantify excitatory synapses on GABAergic neurons in lamina II of the spinal dorsal horn. Confocal images depicting co-staining of GAD67 (green) with the postsynaptic marker PSD-95 (blue) and the presynaptic marker synaptophysin (red). Bottom right, white arrowheads in enlarged boxed areas indicate colocalization of the three antigens. Scale bars: 10 µm (2 µm for enlarged boxes).

In addition we performed immunohistochemical triple staining and confocal microscopy in five animals per group for GAD67 positive neurons in combination with PSD-95, an established marker for excitatory AMPAergic synapses in lamina II [26], [27], and the presynaptic marker synaptophysin (Fig. 5). We used the isoform GAD67 of the GABA synthesizing enzyme glutamic acid decarboxylase (and the promoter for EGFP expression in the transgenic mice) as a marker for inhibitory GABAergic spinal neurons because the intensity of the EGFP labeling was too weak as compared to the synaptic markers. None of the quantification methods used (Mander’s colocalization coefficients or analyzing the relative area of overlaid markers) did reveal any statistically significant differences between sham- and CCI-treated animals in any combination tested (Table 2).

**Table 2:** Quantification of colocalization of GAD67 with the postsynaptic marker PSD-95 and the presynaptic marker synaptophysin in spinal lamina II.

	CCI		Sham	
	ipsilateral	contralateral	ipsilateral	contralateral
Mander’s coefficient				
M_GAD-Synapto_	0.69±0.02	0.69±0.02	0.67±0.02	0.67±0.02
M_Synapto-GAD_	0.29±0.04	0.29±0.03	0.28±0.02	0.27±0.03
M_GAD-PSD95_	0.43±0.04	0.40±0.05	0.50±0.05	0.49±0.04
M_PSD95-GAD_	0.20±0.01	0.22±0.02	0.23±0.01	0.24±0.03
M_Synapto-PSD95_	0.34±0.02	0.36±0.02	0.37±0.01	0.37±0.02
M_PSD95-Synapto_	0.48±0.02	0.51±0.03	0.49±0.01	0.50±0.04
Colocalization (rel. area, %)				
GAD-Synapto	17.02±1.91	17.76±1.77	16.16±1.31	16.36±1.53
GAD-PSD95	9.29±0.62	10.36±1.36	11.19±1.33	11.20±1.07
PSD95-Synapto	21.22±0.92	20.95±1.29	22.62±0.79	22.97±1.18
GAD-Synapto-PSD95	7.52±0.52	8.20±1.02	8.64±0.29	8.76±0.81

Using two different approaches for colocalization analyses (Mander’s colocalization coefficients and analyzing the relative area of combined channels), triple immunofluorescence staining of GAD67 with PSD-95 and synaptophysin revealed no significant difference in any of the parameters tested between sham-treated (n = 5) and CCI (n = 5) animals (two-way ANOVA, for all groups: P>0.05). Data: mean ± s.e.m.

Thus, the present data do not provide any evidence for either a loss of excitatory synapses or a morphological change in dendritic spines of spinal GABAergic neurons in animals with a CCI of the sciatic nerve.

### Reduced transmitter release probability at terminals of Aδ- and C-fibers contacting GABAergic neurons following CCI

**Figure 6:**
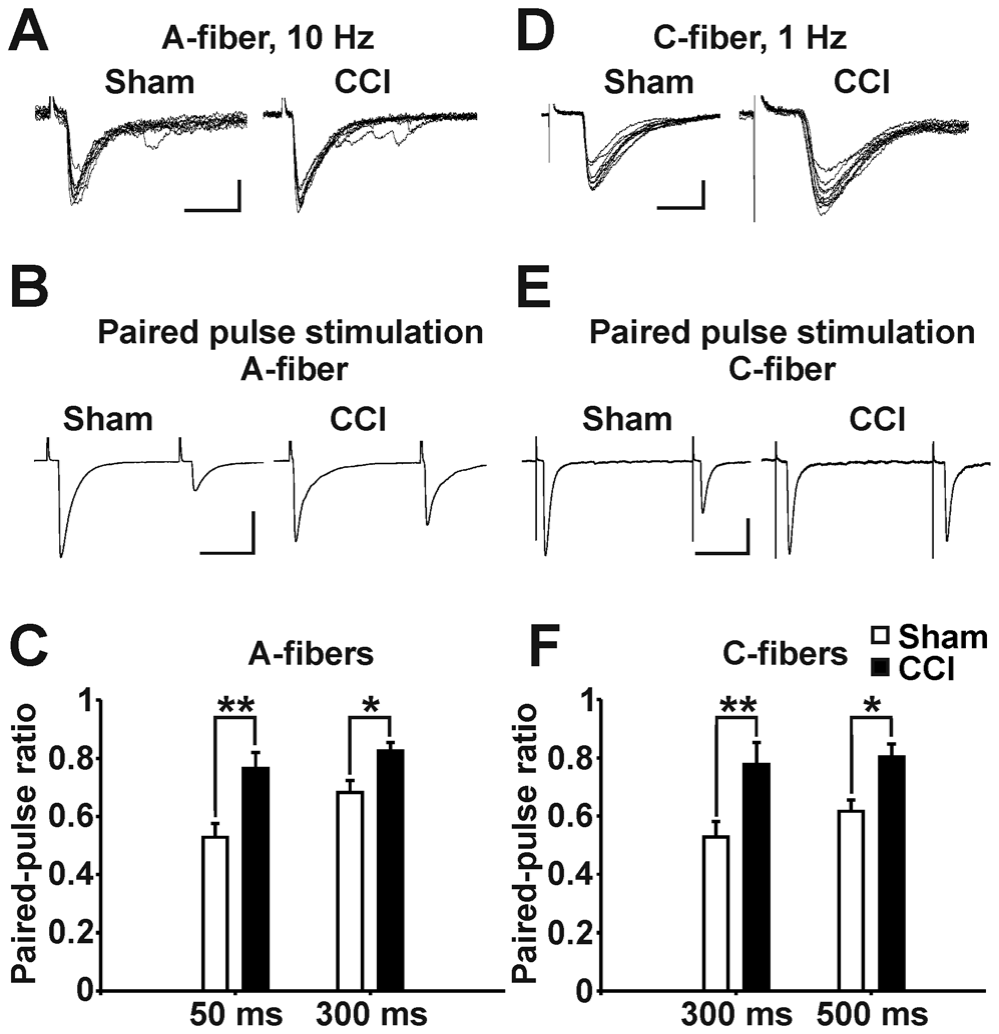
Paired-pulse ratio recorded from EGFP-labeled, GABAergic neurons following dorsal root stimulation is increased after CCI treatment. (**A**) Overlaid monosynaptically evoked Aδ-fiber EPSCs during 10 Hz stimulation (scale bars: 10 ms, 50 pA). (**B**) Averaged traces of paired-pulse recordings following Aδ-fiber stimulation at 50 ms interstimulus interval (scale bars: 20 ms, 100 pA). (**C**) Paired-pulse ratio following Aδ-fiber stimulation was increased in CCI treated mice at 50 ms interstimulus interval (sham n = 19, CCI n = 20, ** P < 0.01, two-way ANOVA) and at 300 ms interstimulus interval (sham n = 21, CCI n = 20, * P < 0.05, two-way ANOVA). Data: mean ± s.e.m. (**D**) Overlaid monosynaptically evoked C-fiber EPSCs during 1 Hz stimulation (scale bars: 10 ms, 50 pA). (**E**) Averaged traces of paired-pulse recordings following C-fiber stimulation at 300 ms interstimulus interval (scale bars: 100 ms, 100 pA). (**F**) Paired-pulse ratio following C-fiber stimulation was increased in CCI treated mice at 300 ms interstimulus interval (sham n = 13, CCI n = 13, ** P < 0.01, two-way ANOVA) and at 500 ms interstimulus interval (sham n = 16, CCI n = 16, * P < 0.05, two-way ANOVA). Data: mean ± s.e.m.

We then tested if the release probability at excitatory synapses contacting GABAergic neurons might be reduced in CCI animals. As an estimate for release probability we evaluated the paired pulse ratio (PPR) of EPSCs evoked from presumably nociceptive Aδ- and C-fiber afferents. Only EPSCs evoked monosynaptically from either Aδ-fiber or from C-fiber stimulation were analyzed (Fig. 6A and D). Paired pulse EPSC recordings were made from 21 EGFP-labeled neurons in sham-treated animals and from 20 EGFP-labeled neurons in CCI animals with monosynaptic input from Aδ-fibers. In addition 16 neurons each in sham-treated and in CCI animals were studied with monosynaptic C-fiber input. The stimulation intensities to induce EPSCs were not significantly different in CCI-treated animals as compared to sham-treated controls for Aδ-fibers (sham: 203±32 µA, CCI: 258±44 µA) or for C-fibers (sham: 1.7±0.2 mA, CCI: 2.1±0.2 mA). All recorded neurons expressed paired pulse depression but at significantly different levels (Fig. 6B and E). The PPRs of both, Aδ- and C-fiber-evoked responses were significantly higher in neurons recorded from CCI animals as compared to controls (Fig. 6C and F). The increase of the PPR in neuropathic animals indicates a decreased release probability at excitatory synapses between Aδ- or C-fibers and spinal GABAergic neurons.

One could speculate that the mean amplitudes of evoked EPSCs could also be decreased in slices taken from animals that underwent a CCI-surgery. This was however not the case: We found no significant differences when analyzing the amplitudes of the first responses evoked by either Aδ- (sham: 207±35 pA; CCI: 189±25 pA) or C-fiber stimulation (sham: 202±33; CCI: 192±62). This finding is not surprising, since the amplitudes of evoked EPSCs do not only depend on the synaptic release probability and stimulus strength but also on variable experimental conditions (e.g. different numbers of active synapses due to inevitable preparational differences, different arborization of dendritic trees within the slice preparation), resulting in a high variance.

### Ca^2+^-dependent signaling in GABAergic neurons presynaptically controls excitatory input

**Figure 7:**
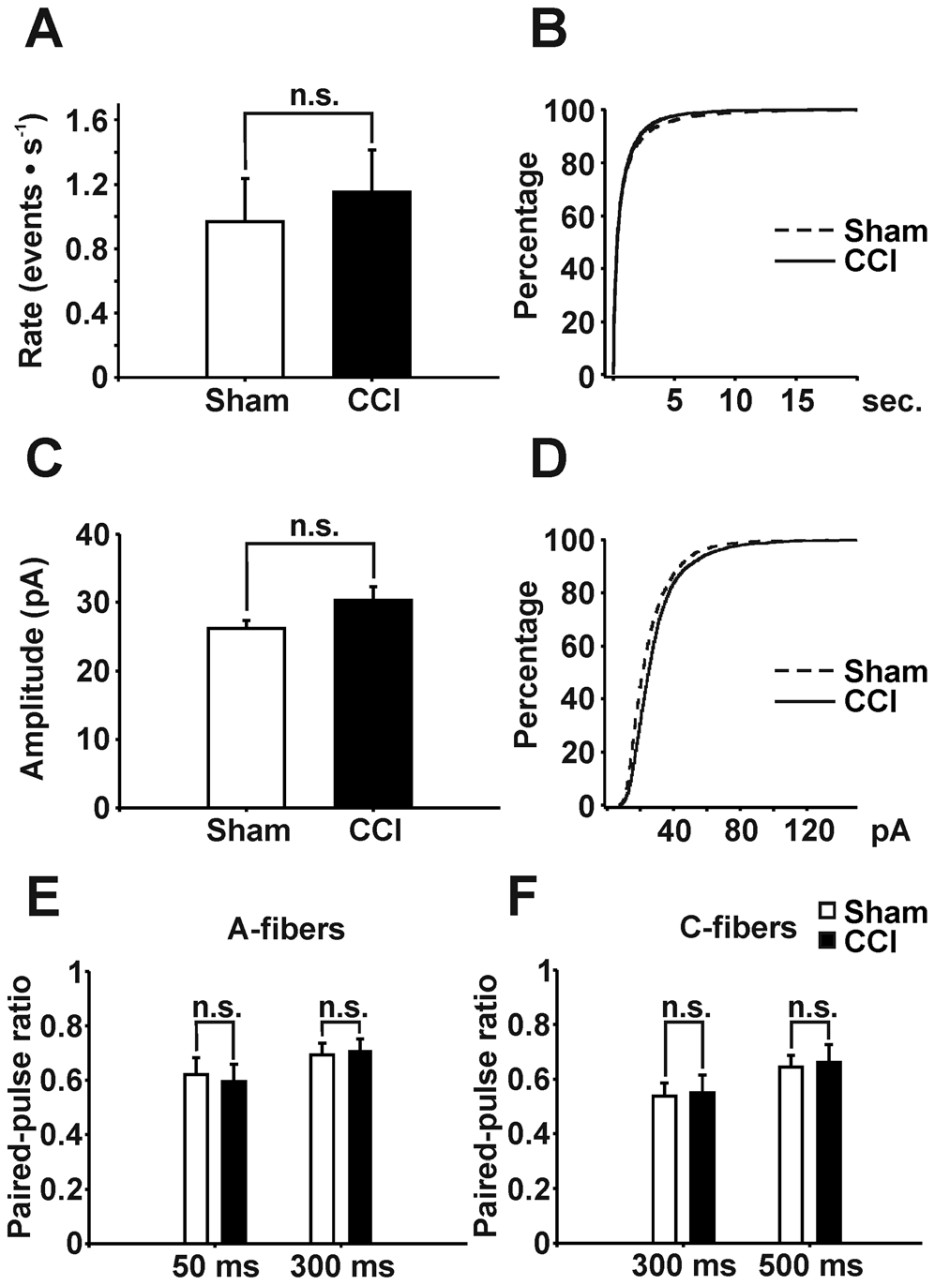
mEPSCs and PPRs recorded from EGFP-labeled, GABAergic neurons with BAPTA included in recording pipette. mEPSCs from GFP-labeled neurons with intracellular BAPTA did not show statistically significant differences between CCI-treated and sham-operated mice in rate (**A**: sham n = 23; CCI n = 21, P>0.1, Mann-Whitney rank sum test) and amplitude (**C**: sham n = 23; CCI n = 21, P>0.1, Mann-Whitney rank sum test) – compare with Fig. 3. B and D: Corresponding cumulative probability plots of inter-event-intervals (**B**) and amplitudes (**D**) of mEPSCs. (**E**) and (**F**) Paired-pulse ratios were not different in CCI-treated mice as compared to sham-operated control mice in none of the interstimulus intervals tested neither following Aδ-fiber stimulation (sham: n = 13, CCI: n = 16; E) nor C-fiber stimulation (sham: n = 19, CCI: n = 12; F) (P>0.1 for all groups, two-way ANOVA). Data: mean ± s.e.m.

CCI-treatment leads to an increase in Ca^2+^ concentration in spinal dorsal horn neurons [28]. We thus asked if a Ca^2+^-dependent synthesis and/or release of an inhibitory retrograde messenger in GABAergic neurons could account for the reduced global excitatory drive [29]. We added the Ca^2+^ chelator BAPTA to the recording pipette to prevent any rise in intracellular Ca^2+^ ions of the GABAergic neurons under study. Under this condition, neither mEPSC rate (Fig. 7A and B) nor amplitude (Fig. 7C and D) nor the PPRs of both Aδ- (Fig. 7E) and C-fiber-evoked (Fig. 7F) responses were significantly different between CCI- and sham-treated mice.

In order to test, if the mEPSC rate in CCI-treated mice increases throughout recording reflecting the effect of a slowly increasing intracellular BAPTA concentration, we calculated the instantaneous frequency of both groups. Recording of mEPSCs started one minute after establishing whole-cell configuration and stopped at minute 10. However, the slope (a) of instantaneous frequency was not different between groups (CCI: a (median, 25%, 75%) = –0.00240, –0.00540, 0.00520; Sham: a (median, 25%, 75%) = –0,00430, –0,0101, 0,00410; P>0.05; Mann-Whitney rank sum test).

These findings indicate that a rise in postsynaptic Ca^2+^ concentration is necessary to presynaptically depress excitatory synaptic drive to GABAergic neurons in CCI-treated animals suggesting the involvement of an inhibitory retrograde messenger.

Various classes of molecules have been found to act as retrograde messengers inhibiting the release of neurotransmitters. This includes lipid-derived messengers such as endocannabinoids acting on cannabinoid-1 (CB_1_) receptors, gases such as nitric oxide (NO) and conventional neurotransmitters including GABA acting on GABA_B_ receptors [29]–[34]. We next explored these possibilities.

### Endocannabinoids acting on CB_1_ receptors do not depress excitatory drive to GABAergic neurons

We first tested if endocannabinoids are tonically released by GABAergic neurons and depress excitatory drive to GABAergic neurons via the CB_1_ receptor. If so, one would expect that blocking CB_1_ receptors would lead to an increase in mEPSC rate. Neither in sham-treated animals nor in CCI-mice mEPSCs were affected by CB_1_ receptor blockade with AM 251 (Table 3). Likewise, bath application of CB_1_ receptor agonist ACEA failed to affect mEPSC rates in slices from naïve animals. Thus endocannabinoids acting on presynaptic CB_1_ receptors are not the cause for reduced excitatory drive to GABAergic neurons in CCI-operated mice.

**Table 3:** Bath application of drugs did not change mEPSC rates.

Treatment	Drug	Concentration	mEPSC rate (% of control)	n
Sham	-	-	86±20%	9
CCI	-	-	88±4%	4
Sham	AM 251	5 µM	89±9%	8
CCI	AM 251	5 µM	98±20%	8
Naïve	ACEA	1 µM	105±26%	4
Naïve	GABA	10 µM	89±16%	4
Naïve	SNAP	200 µM	101±16%	5

mEPSC rates after drug application were normalized to preapplication recording periods. No significant changes were observed (one-way ANOVA, P>0.1). Data: mean ± s.e.m.

### Neither GABA nor nitric oxide modulate global excitatory drive to GABAergic neurons

We then tested if bath application of putative retrograde messengers GABA, or the NO-donor SNAP is capable of reducing mEPSC rates in normal animals. Bath application of neither GABA (10 µM, n = 4) nor SNAP (200 µM, n = 5) had any effect on mEPSC rates (Table 3). Thus, neither GABA nor NO is likely the retrograde messenger that may reduce global excitatory drive of GABAergic neurons.

### c-Fos activation following noxious thermal stimulus

**Figure 8:**
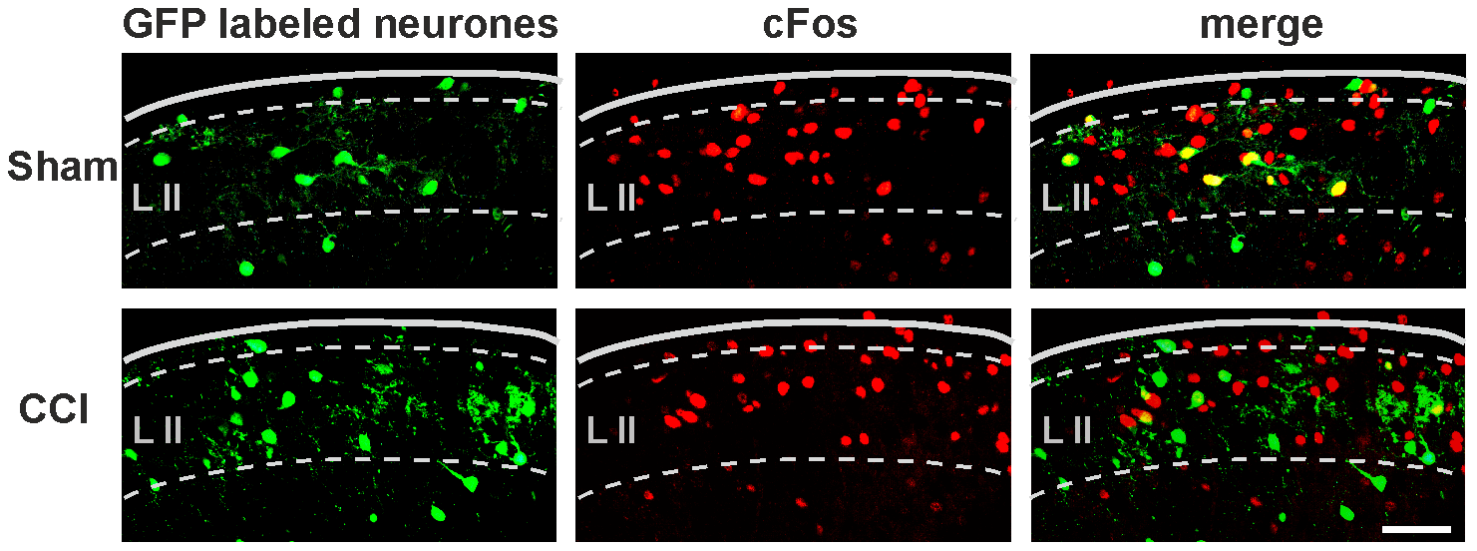
c-Fos expression in GABAergic neurons. Upper row: c-Fos expression after noxious heat stimulus in a sham-treated mouse colocalizes with EGFP-labeled GABAergic neurons. Lower row: In a CCI-operated mouse c-Fos can be detected in fewer EGFP-labeled neurons as compared to a sham-treated mouse. (scale bar: 50 µm, left  =  lateral, right  =  medial) Border between dorsal horn and white matter is indicated by a solid line, lamina borders by a dashed line. Lamina II is indicated by L II.

We next asked if the reduced excitatory drive from primary afferent nociceptive nerve fibers onto spinal GABAergic neurons in slices obtained from CCI-animals has any functional meaning *in vivo*. According to our hypothesis noxious stimulation should activate fewer GABAergic neurons in CCI-treated animals as compared to sham-treated animals. We used the expression of c-Fos protein as a marker for neuronal activity [35]. In CCI-treated animals the number of EGFP-labeled neurons was not different as compared to sham-treated mice. A noxious stimulation induced, however, c-Fos in significantly fewer GABAergic neurons in the medial half of lamina II in the CCI group as compared to controls (Table 4 and Fig. 8). These findings indicate that in neuropathic animals noxious stimuli indeed fail to activate as many spinal GABAergic neurons as they normally do in control animals.

**Table 4:** c-Fos expression in GABAergic neurons following a noxious heat stimulus was reduced in CCI-treated mice.

	Percentage of EGFP-labeled neurons	Total neuronal c-Fos expression in medial lamina II	c-Fos expression in non-EGFP neurons	c-Fos expression in EGFP-labeled neurons
Sham (n = 6)	15.5±1.5%	28.5±2.4%	25.6±3.3%	40.1±1.4%
CCI (n = 6)	14.6±1.4%	26.5±1.7%	25.0±2.8%	25.2±1.5%
Unpaired Student’s t-test	P>0.1	P>0.1	P>0.1	P < 0.001

Percentage of EGFP-labeled neurons was not changed in CCI-treated animals. Following a noxious heat stimulus the total amount of neuronal c-Fos expression and c-Fos expression in non-EGFP neurons was not significantly different between groups. However, c-Fos expression in EGFP-labeled neurons was reduced in CCI-operated as compared to sham-treated animals. Data: mean ± s.e.m.

The overall c-Fos expression as well as the expression in non-EGFP neurons only was not different between groups. Since non-EGFP neurons constitute a very heterogeneous group including excitatory and inhibitory, even GABAergic neurons, we cannot draw any firm conclusion from this finding.

## Discussion

The present study revealed a novel mechanism of impaired spinal inhibition likely contributing to neuropathic pain: the global downregulation of excitatory synaptic drive to GABAergic neurons. Furthermore, the excitatory drive from Aδ- and C-fibers was reduced thus causing an impaired recruitment of inhibitory neurons during nociception. The resulting attenuation of feedforward inhibition may open a “spinal gate” of nociception as illustrated in Fig. 1B, thereby contributing to pain amplification in neuropathic animals and perhaps in pain patients.

Spinal GABAergic neurons are predisposed to function as gatekeepers for nociception. About 30% of the interneurons located in spinal lamina II are GABAergic [36], [37]. Blocking spinal GABA receptors lowers pain thresholds [3], and spinal application of GABA receptor agonists increases pain thresholds in pathological pain states such as neuropathic pain [4], [5].

A number of potential mechanisms that could lead to impaired GABAergic inhibition in nociceptive pathways have been explored. For example, cell death of spinal inhibitory neurons has been demonstrated in several animal models for neuropathic pain [38]. Other studies have, however, shown that the symptoms of neuropathic pain also arise in the absence of any detectable cell death [39], [40]. Previous findings from our group suggest that the excitability of spinal GABAergic neurons is unchanged in animals with CCI [8]. GABAergic output is impaired in animals with a peripheral nerve injury, as demonstrated by a reduction of primary afferent evoked inhibitory postsynaptic currents [6]. In addition, CCI has been shown to lead to a shift of the anion gradient in some lamina I neurons. Thereby, the action of GABA is reduced and shifted towards excitation in neuropathy [7].

Here we studied the excitatory input to spinal GABAergic neurons which were identified by the expression of EGFP under the promoter of GAD 67. This labels a representative population of GABAergic neurons with respect to their electrophysiological, neurochemical and morphological characteristics [16], [41]. CCI induced a significant decrease of mEPSC rate but not amplitude in spinal lamina II GABAergic interneurons in the present study. This demonstrates that CCI leads to a decrease in global excitatory drive to identified GABAergic neurons. Our data are in line with other findings showing a CCI-induced decrease of mEPSC rate in presumably inhibitory interneurons, as judged by their action potential firing pattern and cell morphology, whereas other spinal neuronal subgroups exhibited enhanced mEPSC rates [42], [43].

The rate of mEPSCs reflects the release probability of neurotransmitter from presynaptic terminals given the number of synapses is constant [44]. In the central nervous system the number of active synapses can change e.g. after induction of synaptic long-term potentiation [45]. Thus, the reduced mEPSC rate in GABAergic neurons found in this study could either be due to a reduced release probability of neurotransmitter from presynaptic terminals or to a reduced number of excitatory synapses at GABAergic neurons. Assuming that the majority of excitatory synapses terminate on dendritic spines [23], [24], a loss of spines should reflect decreased excitatory input [46]. Dendritic spines are highly plastic and have been shown to alter in size and shape within minutes in response to changes in the pattern or the intensity of synaptic input (for review see [25]). Accordingly, the quantification of dendritic spines can be used as a measure of the number of excitatory synapses. In contrast to some regions in the brain GABAergic neurons in the spinal cord do form dendritic spines [47]. In our sample of 3D-reconstruction all GABAergic neurons (n = 25) displayed dendritic spines. These neurons represent a random selection of EGFP-labeled neurons, which was not used for electrophysiology, since calcein filling reduced the quality of the recordings. We therefore could not quantify the proportion of spine bearing neurons in our electrophysiological experiments. In parasagittal slices, neither density nor length, diameter, surface, or volume of dendritic spines on spinal lamina II GABAergic neurons were altered in CCI-treated animals as compared to sham-operated mice. Dendritic spines on GABAergic neurons in lamina II of the spinal cord have not been studied extensively yet. Furthermore our results do not exclude that the number of excitatory synapses contacting dendritic shafts [48] might have dropped in CCI-treated animals. To also account for these synapses we performed confocal microscopy of immunohistochemical stainings for GAD67 neuronal structures, PSD95, a marker for excitatory synapses [26], [27], and the presynaptic maker synaptophysin. These stainings also revealed no differences between CCI-treated and control mice. Thus, we found no evidence that the number of excitatory synapses on spinal GABAergic neurons is altered in neuropathic animals.

We next tested if release probability of neurotransmitter from these primary afferent fibers terminating onto lamina II GABAergic neurons may be changed. An inverse relationship exists between the probability of neurotransmitter release and the PPR [49], [50]. In CCI-treated animals the PPR calculated for synapses of primary afferent Aδ- or C-fibers with spinal GABAergic neurons was always higher as compared to sham controls suggesting a reduced release probability. One can, however, not fully exclude a potential postsynaptic contribution to changes in PPR [51]. Collectively, both phenomena PPR and mEPSCs point to a decrease in release probability under different experimental conditions (i.e. action potential dependent and action potential independent release of neurotransmitter).

A potential explanation for a reduced release probability at synapses is the release of a retrograde, inhibitory messenger by the postsynaptic neuron. This often requires a rise in postsynaptic Ca^2+^ ion concentration (for review see [29]). In animals with CCI the intracellular Ca^2+^ concentrations in neurons of the superficial and deep spinal dorsal horn are indeed enhanced [28]. Our data now demonstrate that an enhanced Ca^2+^ ion concentration in GABAergic neurons is indispensable for the depression of their global excitatory drive. The requirement of postsynaptic Ca^2+^ rise in combination with a reduced release probability strongly suggests the involvement of a retrograde inhibitory messenger.

Possible candidates include endocannabinoids, which are released following intracellular Ca^2+^ rise and act on presynaptic CB_1_ receptors thereby reducing neurotransmitter release at excitatory synapses [31]. CB_1_ receptors are abundantly expressed in the spinal cord dorsal horn [52], [53]. Our data demonstrate, however, that neither the CB_1_ receptor agonist ACEA nor the receptor antagonist AM 251 exerted any detectable effects on the mEPSC rates. GABA, which can act as a retrograde messenger as well inhibiting presynaptic neurotransmitter release via GABA_B_ receptors [30], or NO, a retrograde messenger in the CNS shown to increase [33], [34] or in some cases decrease [32] transmitter release, did not alter mEPSC rates either. This is in line with findings that nitric oxide synthase is only expressed in a small subset of EGFP-expressing GABAergic neurons in the spinal superficial laminae [16], [41]. The present data thus make it unlikely that endocannabinoids acting on CB_1_ receptors, GABA acting on GABA_B_ receptors or NO account for the diminished global excitatory drive onto GABAergic neurons in neuropathic animals. Future studies will be needed to clarify the nature of the retrograde signaling.

We further showed that activation of GABAergic neurons in lamina II of the spinal dorsal horn is significantly impaired in CCI-animals as compared to controls. We used the expression of c-Fos protein as a marker for nociceptive activity in spinal dorsal horn at the single cell level [35]. After a CCI-induced initial rise c-Fos expression returns to normal in laminae I and II within 10 to 14 days [54], [55]. In addition, noxious heat-evoked c-Fos expression in laminae I and II are not different in control versus CCI-treated animals when the global expression is assessed [56]. We performed our experiments on day eleven post-surgery and our results on the global c-Fos expression in lamina II are in full agreement with those previous studies. Only when we quantified c-Fos expression selectively in identified GABAergic neurons, we found a reduced expression in the CCI-treated animals indicating that activation of spinal GABAergic neurons is severely reduced in neuropathic animals.

In summary, we showed that peripheral neuropathy triggers Ca^2+^-dependent signaling pathways in spinal GABAergic neurons, resulting in a global downregulation of their excitatory drive. In addition, monosynaptic excitation of GABAergic neurons by Aδ- and C-fibers during noxious stimulation is reduced, thus impairing normal feedforward inhibition and opening a spinal gate for nociceptive messages.
